# Original Letters of Dr. Mead

**Published:** 1803-07-01

**Authors:** R. Mead

**Affiliations:** Ormond Street


### ORIGINAL LETTERS OF DR. MEAD.

*The Two following Letters of* Dr. Mead, *never before published, and written about eighty years ago, may perhaps be worth inserting in the Medical and Physical Journal; if not on account of the importance of the matter they contain, at least as literary curiosities.*

To Dr. DEACON, at Manchester.

SIR,

I Received your's concerning Mr. Clayton's case yesterday, and have writ to him, as you desired, by this post. I have assured him there is no danger. You will see my letter. I am by this to acknowledge the favour of your's of the 15th. As to your patient with the fluor albus, I don't know any thing so effectual as an injection of aq. camphorat. batean. lib. j. mell. Ægyptiac. ℥iij. made use of morning ing and evening. Aq. benedict. comp. batean. is good, as is Bristol water. Pills made of alum melted ℥iij. sang. dracon. ℥j. taken ℈j. ter die are serviceable.

*Ormond Street,*

*December* 24, 1724. I am, &c. R. MEAD.

---

### To Dr. DEACON, at Manchester.

DEAR SIR,

I HAVE the favour of your's concerning Mr. Darbyshire's case, which is indeed very bad. I must freely observe to you, that I have always experienced that mercury in no shape whatsoever will agree with cancerous cases, which are always exasperated by it; that is, while the cancerous tumour remains in any part; for when that is cut off, I have known a salivation, if the patient has been put into it as soon as the wound begins to digest, and it has been continued till the part is quite healed, to have done prodigious service, and the humour has never returned again. The mercurius alcalisatus frequently raises a salivation, and I suppose it has done so in the present case the more readily from the acrimony of the humours of the body. It is certain that the actual cautery is rightly applied in a cancer; any common caustic, by reason of the sharp salts in its composition, are not agreeable to the corroding salts in the tumour. What therefore can now be done is, I think, if possible, to stop the salivation; as the mercury took this turn from the acrimony of the juices of the body, so I imagine that it goes on so obstinately from the same cause. I would continue to keep the body lax, by gentle purges; infus. sen. cum mann. &c. every third or fourth day; the intermediate days, I would give aq. hord. et lact. vacc. aa. ℔ß. (edulcorat) with fl. sulphur. ʒß. or more, if the stomach will bear it, twice a day. Adstringent gargles must be continued. The diet should be very much upon milky things. If by this means the patient can be brought into that condition as to bear the cautery, it should certainly be applied, and he should then live upon a vegetable and milky diet, and drink freely of a decoction of sars. chinc with milk; and take pulv. milleped. ʒß. twice a day. I shall be glad to hear how you proceed, and am always,

*Ormond Street,*

*March* 20, 1735-6. Your's, &c. R. MEAD.

